# Sinus Mucosal Damage Triggered by Synthetic or Xenogeneic Bone Substitutes: A Histological Analysis in Rabbits

**DOI:** 10.3390/jfb13040257

**Published:** 2022-11-19

**Authors:** Yuki Omori, Daniele Botticelli, Stefano Migani, Vitor Ferreira Balan, Eduardo Pires Godoy, Samuel Porfirio Xavier

**Affiliations:** 1 Department of Oral Implantology, Osaka Dental University, 8-1 Kuzuhahanazonocho, Osaka 573-1121, Japan; info@omori-dent.com; 2 ARDEC Academy, Viale Giovanni Pascoli 67, 47923 Rimini, Italy; dott.miganistefano@gmail.com; 3 Department of Oral and Maxillofacial Surgery and Periodontology, Faculty of Dentistry of Ribeirão Preto, University of São Paulo, Av. do Café-Subsetor Oeste-11 (N-11), Ribeirão Preto 14040-904, Brazil; vitor.balan@usp.br (V.F.B.); spx@forp.usp.br (S.P.X.); 4 Department of Oral Biology, Faculty of Dentistry of Ribeirão Preto, University of São Paulo, Ribeirão Preto 14040-904, Brazil; eduardo.godoy@usp.br

**Keywords:** animal study, sinus floor elevation, bone healing, Schneiderian membrane, histology, sinus mucosa perforation

## Abstract

Background: It has been shown in rabbit models that the sinus mucosa in contact with graft particles might experience a progressive thinning and perforations. The phenomenon depends on the graft used. Hence, the aim of the present study was to compare the damaging effects of a synthetic of a xenogeneic graft. Methods: Forty New Zealand rabbits received a bilateral sinus elevation. Both sinuses of twenty rabbits were grafted with a biphasic 60% hydroxyapatite and 40% β-tricalcium phosphate while the other twenty received a deproteinized bovine bone mineral graft. Thinned sites (<40 µm) and perforations on the mucosa in contact with graft particles were evaluated after 2 and 10 weeks (ten animals each period). The width of the pseudostratified epithelium was also measured as control. Results: After 2 weeks of healing, 61 thinned sites were detected in the Synthetic group and 49 in the Xenogeneic group. After 10 weeks, the number of thinned mucosae increased to 79 sites in the Synthetic group (*p* = 0.222 between periods), and to 114 sites in the Xenogeneic group (*p* = 0.030 between groups; *p* = 0.001 between periods). Perforations were few in the 2-week period, two in two sinuses out of 20 in the Synthetic group, and four in two sinuses out of 20 in the Xenogeneic group (*p* = 0.721). In the 10-week period, the perforations increased to eight in the Synthetic group, distributed in six sinuses out of 20, and to sixteen in the Xenogeneic group, distributed in 11 sinuses out of 20 (*p* = 0.082). The pseudostratified epithelium presented a reduced width at the thinned sites. Conclusions: The contact with synthetic or xenogeneic grafts will induce thinning and possible perforations of the sinus mucosa. This effect will increase over time, and it is stronger at the xenogeneic than the synthetic graft.

## 1. Introduction

The lateral approach for sinus floor elevation has been well documented in literature, resulting in a high success rate [1,2]. Like any surgical technique, this can also present complications, the most frequent being the perforation of the sinus mucosa [3,4]. The perforations might occur during sinus mucosa elevation, grafting procedure, or implant installation [5,6,7]. Perforations might heal spontaneously [6], and it was not considered a risk factor for dental implant survival in a systematic review with meta-analysis [8]. Nevertheless, sinusitis has been associated with biomaterial extruded into the sinus [8,9,10,11,12], an event that induced some surgeons to remove the extruded graft from the sinus cavity [11,12].

However, it has recently been demonstrated in experiments in rabbits that the sinus mucosa in contact with graft particles might become thinner over time and eventually perforate [13,14,15]. It has also been shown that the number of thinned and perforated sites depends on the characteristics of the biomaterial in contact with the sinus mucosa [13,15]. In an experiment in rabbits, sinus augmentation was performed bilaterally using either autogenous bone or a deproteinized bovine bone mineral [13]. Implants were also installed simultaneously. After 40 days of healing, due to the higher resorption rate of the autogenous compared to the xenograft grafts, only one thinned site (width <40 µm) and no perforations were found at the sinus mucosa in contact with autogenous grafts, while 96 thinned sites and three perforations were observed at the sinus mucosa in contact with the xenograft particles. A direct contact with the implant apex and threads may also result in perforations of the sinus mucosa [13,16]. Due to the results of these reports, further evaluations should be performed aiming to find the biomaterial that elicits the lower damage to the sinus mucosa. Hence, the aim of the present study was to compare the damaging effects on the sinus mucosa of a synthetic and a xenogeneic graft.

## 2. Materials and Methods

### 2.1. Ethical Statements

Two experiments on sinus floor augmentation in rabbits using a synthetic or a xenogeneic graft were evaluated. The experimental protocols were approved by the Ethical Committee of the Faculty of Dentistry of Ribeirão Preto, University of São Paulo (Synthetic study: 2018.1.454.58.2, approved on 19 September 2018; Xenogeneic study: protocol No 2018.1.10.58.7, approved on 21 March 2018). The article was written according to the ARRIVE guidelines. The Brazilian rules for animal care were accurately followed.

### 2.2. Study Design

Maxillary sinus augmentation was carried out bilaterally in rabbits. A biphasic hydroxyapatite and beta-tricalcium phosphate (HA β-TCP) in the synthetic study, and a deproteinized bovine bone mineral (DBBM) in the xenogeneic study, were used as grafts. Both biomaterials were treated with argon plasma at the test sites while no treatments were provided to the grafts used at the control sites. Sinus mucosa thinning and perforations were assessed. The histomorphometric data describing the healing within the elevated regions were reported elsewhere [17,18].

### 2.3. Experimental Animals

In each experiment, twenty albino New Zealand rabbits, ~3.5–4 kg of weight and 5–6 months old, were used. Two groups composed of 10 animals were obtained in each experiment and euthanized after 2 or 10 weeks from surgery, respectively.

### 2.4. Biomaterials

Two grafts have been evaluated: a synthetic group: GUIDOR Calc-i-oss CRYSTAL+ (Sunstar, Etoy, Switzerland) composed of 60% hydroxyapatite and 40% β-tricalcium phosphate irregular-shaped granules with dimensions of 0.450–1.0 mm; and a xenogeneic group: Bio-Oss^®^ (granules 0.250–1.0 mm; Geistlich Biomaterial, Wolhusen, Switzerland) composed of a bovine inorganic, porous hydroxyapatite from cancellous bone deproteinized at a temperature of 300 °C.

### 2.5. Sample Size

The sample size was determined based on the number of thinning mucosa sites in contact with the biomaterials after 40 days of healing reported by a previously discussed study in rabbits [13]. The determined effect size was 2.983. Applying an α = 0.05, a power of 0.9, four animals each group were obtained. A slower resorption of the synthetic biomaterial used in the present study compared to autogenous bone used in that study used for calculation [13] might increase the number of thinning sites, decreasing the difference with the xenogeneic graft. Hence, ten instead of four animals for each group were considered sufficient to reject the null hypothesis that the population means of the two groups are equal.

### 2.6. Randomization and Allocation Concealment

The randomization plan was applied originally for the argon plasma treatment in both studies. The histological assessor was not informed before measurement about a possible comparison of the data between the two different studies. Mean values or sum of the data were used for the two sinuses of each rabbit so that the animal was the statistical unit used for analyses.

### 2.7. Clinical Procedures

The anesthetic and surgical procedures were similar in both experiments. Briefly, acepromazine (1.0 mg/kg, Acepran^®^, Vetnil, Louveira, São Paulo, Brazil) subcutaneously and xylazine (3.0 mg/Kg, Dopaser^®^, Hertape Calier, Juatuba, Minas Gerais, Brazil) and ketamine hydrochloride (50mg/kg, Ketamin Agener, União Química Farmacêutica Nacional S/A, Embu-Guaçú, São Paulo, Brazil) intramuscular were administrated. The rabbit’s muzzle was shaved and disinfected. An incision was performed in the midline of the nasal dorsum by expert surgeons, and the nasal bone was exposed. Osteotomies were prepared using trephines and drills. A small screw was applied in the nasal-incisal suture as a reference for histological process. The sinus mucosa was elevated, and the biomaterial was grafted into the sub-antral spaces. The access windows were subsequently covered using a collagen membrane (Bio-Gide, Geistlich Biomaterial, Wolhusen, LU, Switzerland). For more details, see the previous published articles [17,18].

### 2.8. Euthanasia

The rabbits were first anesthetized, and euthanasia was performed in a closed transparent acrylic box containing gas carbon dioxide (CO_2_) or an overdose of sodium thiopental (1.0 g, 2 mL, Thiopentax^®^, Cristália Produtos Químicos Farmacêuticos, Itapira, São Paulo, Brazil).

### 2.9. Housing and Husbandry

The animals were kept in individual cages in a climatized room with access to food and water ad libitum. The biological functions and the wounds were checked daily by specialized operators for the whole period of the experiment. A prophylactic dose of oxytetracycline dehydrate (40 mg/kg, IM, Terramicina LA, Zoetis Indústria e Produtos Veterinários, Campinas, São Paulo, Brazil) and, postoperatively, ketoprofen (3.0 mg/kg, IM., Ketofen 1%, Merial, Monte-Mor, Sao Paulo, Brazil) and tramadol hydrochloride (Tramadol 2%, 1.0 mg/kg, SC., Cronidor, Agener União Saúde Animal, Apucarana, Parana, Brazil) for 2 days were administrated.

### 2.10. Histological Preparation

After fixation with formalin, the biopsies were dehydrated and then embedded in resin (LR White™ hard grid, London Resin Co., Ltd., Berkshire, UK) and polymerized. Two grounds sections representing the central region of the sinuses were obtained using a cutting and grinding equipment (Exakt^®^, Apparatebau, Norderstedt, Germany). The sections were stained with either Stevenel’s blue and alizarin red or toluidine blue.

### 2.11. Calibration for Histometric Evaluations

All measurements were carried out by an assessor (S.M.) after a training with an expert (D.B.). The intra-rater reliability in the measurements of the sinus mucosa width and perforation dimensions was K > 0.90.

### 2.12. Histological Analyses

The pristine mucosa was measured at the medial and lateral sinus walls in regions not included in the elevated area. A mean value of the two measurements was used for comparisons with the thinned sites.

The number and the width of the elevated sinus mucosa in close contact with the graft granules was measured, and all measurements <40 µm were recorded. The number and dimensions of the sinus mucosa perforations at the graft granules were also assessed.

### 2.13. Experimental Outcomes and Statistical Methods

The Wilcoxon test was used for dependent variables while a Mann–Whitney test was applied for independent variables. The software Prism 9.4.1 (GraphPad Software, LLC, San Diego, CA, USA) was used for statistical analyses.

## 3. Results

### 3.1. Clinical Outcomes

One sinus of the 2-week xenogeneic group presented a small perforation of ~0.5 mm that was protected with a small piece of collagen membrane. No complications were observed during healing in any group. At the time of histomorphometric analysis presented in a previous paper, one biopsy of the 10-week synthetic groups was not available for analysis due to technical problems. However, for the present analysis, the histological slide was available for analysis allowing n = 10 for all groups and periods.

### 3.2. Descriptive Histological Evaluation

**Figure 1:**
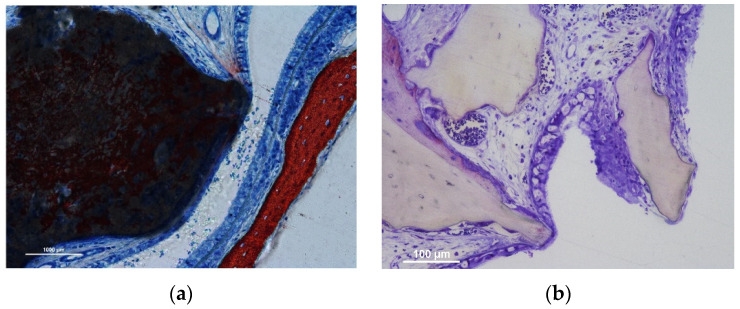
(**a**) Synthetic site: Stevenel’s blue and alizarin red stain. (**b**) Xenogeneic site: toluidine blue stain. Note the progressive decrease in width of both sinus mucosae and pseudostratified epithelia. A loss of cilia is evident in the thinnest sites on both biomaterials. While the process of resorption has a minimal impact on the xenogeneic graft, the synthetic graft has undergone a process already described as an interpenetrating bone network [18] characterized by concurrent bone formation within the biomaterial structure during its resorption.

Several thinned mucosa sites (<40 µm) were identified in both groups (Figure 1a,b).

**Figure 2:**
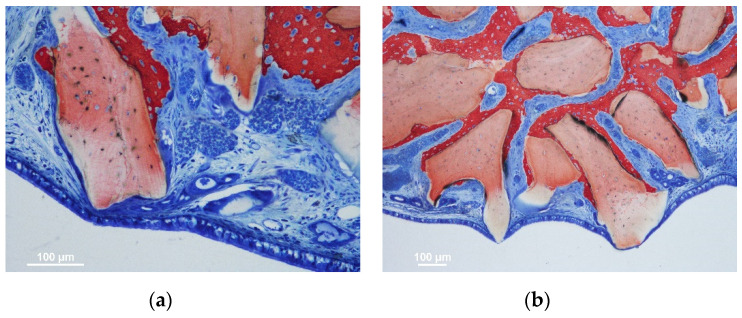
(**a**,**b**) Xenogeneic sites presenting displacement of mucosal glands and vessels. A progressive thinning of sinus mucosa and pseudostratified epithelium was observed in several sites in close contact with the biomaterial granules. Note the new bone apposition on the biomaterial surface in sites opposite to the sinus mucosa.

The number of such sites was slightly higher in the synthetic compared to the xenogeneic groups in the 2-week period. However, that condition was reversed in the 10-week period due to a vast increase in the xenogeneic group of the number of thinned sites. The thinned sites were characterized by a tight contact towards the biomaterial granules resulting in a progressive damage to the sinus mucosa. In the initial stages, only the lamina propria was involved in histological modification, presenting vessels and mucosal glands displaced and deformed as if pressed against the granules surface (Figure 2a,b).

In the more advanced stages, the pseudostratified epithelium decreased in width, presenting a progressive loss of globet cells and cilias. In some cases, only a very thin layer of epithelial cells or connective tissue were limiting the direct continuity with the sinus cavity (Figure 1 and Figure 2). The thinned sites were generally devoid of inflammatory infiltrates.

**Figure 3:**
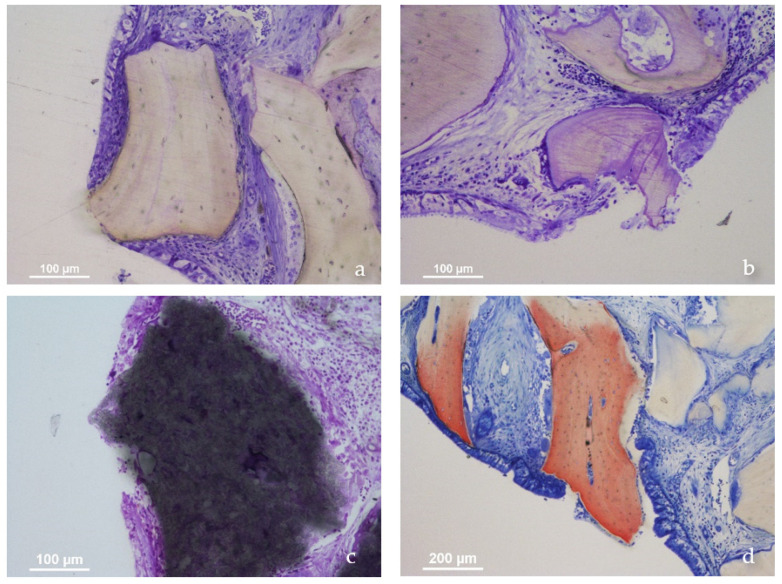
(**a**,**b**,**d**) Xenogeneic sites; (**c**) synthetic site. Note a tapered epithelium bordering the exposed surface of the granules in a manner to maintain the integrity of the underlying tissues.

Few perforations were observed in the 2-week period, while the number was found increased considerably in the 10-week period, especially in the xenogeneic group. A tapered epithelium was generally bordering the exposed surface of the granules in a manner to maintain the integrity of the underlying tissues (Figure 3a–d). Several sites presented few inflammatory cells, while, in other cases, the infiltrates were more evident.

### 3.3. Histometric Assessments

**Figure 4:**
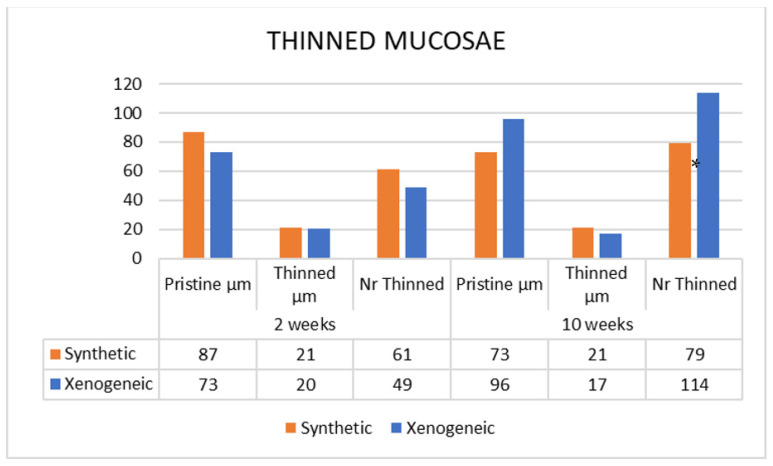
Number of thinned sites in the synthetic and xenogeneic groups after 2 and 10 weeks of healing. * = *p* < 0.05.

**Figure 5:**
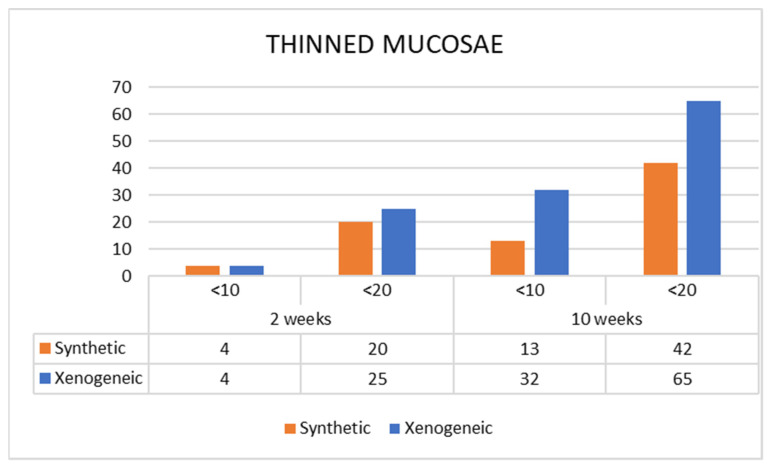
Number of sites presenting sinus mucosa width <10 µm or <20 µm in both synthetic and xenogeneic groups after 2 and 10 weeks of healing.

The mean width of the pristine sinus mucosa ranged between 73 µm and 96 µm. None of the pristine mucosa evaluated presented a width <40 µm. After 2 weeks of healing, 61 thinned sites were detected in the Synthetic group, presenting a mean width of 21 µm (Figure 4). In the Xenogeneic group, the sites were 49 (*p* = 0.537 between group) with a mean width of 20 µm (*p* = 0.561). After 10 weeks, the number of thinned mucosae increased to 79 sites in the Synthetic group (*p* = 0.222 between periods) maintaining the same mean width, and to 114 sites in the Xenogeneic group (*p* = 0.030 between groups; *p* = 0.001 between periods) presenting a mean width of 17 µm (*p* = 0.070 between groups; *p* = 0.105 between periods). The number of sites presenting a width <10 µm and <20 µm increased between 2 and 10 weeks (Figure 5).

**Figure 6:**
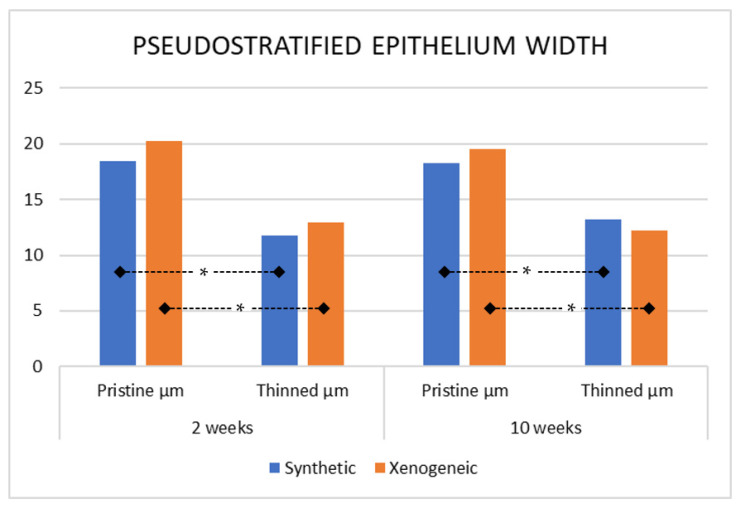
Pseudostratified epithelium width at the pristine not elevated region and at the thinned sites. * = *p* < 0.05.

The pseudo-stratified epithelium at the thinned sites was also thinner compared to the pristine mucosa. The mean values of the pristine epithelium range between 18–20 µm while those at the thinned sites were 12–13 µm (Figure 6). The cilia were not considered in the measurements. The differences between pristine and thinned mucosa were statistically significant for both groups and periods (*p* < 0.01) while no differences were found between groups.

**Figure 7:**
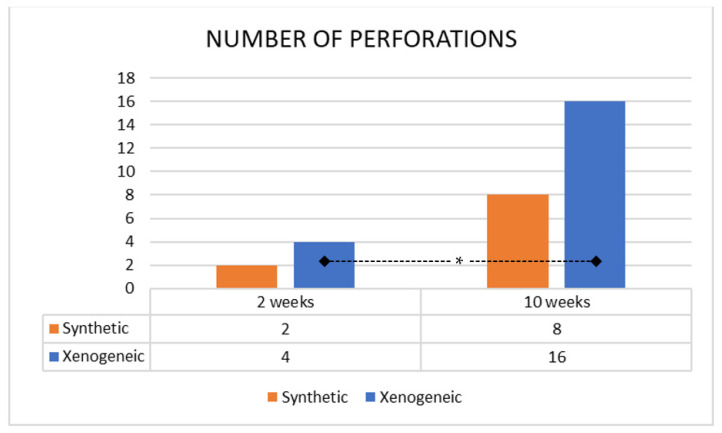
Number of perforations in both the synthetic and xenogeneic groups after 2 and 10 days of healing. * = *p* < 0.05.

There were few perforations were in the 2-week period (Figure 7), two in two sinuses in the Synthetic group, and four in two sinuses in the Xenogeneic group (*p* = 0.721). In the 10-week period, the perforations increased by 4 in both groups so that eight perforations were detected in the Synthetic group, distributed in 6 sinuses out of 20, and sixteen in the Xenogeneic group distributed in 11 sinuses out of 20 (*p* = 0.082). The difference between periods was statistically significant only for the Xenogeneic group (*p* = 0.020). The mean dimension of the perforations was 171 ± 133 µm for the Synthetic group and 123 ± 47 for the Xenogeneic group.

## 4. Discussion

The aim of the present study was to compare the damaging effects on the sinus mucosa after sinus augmentation produced by synthetic or xenogeneic biomaterials used as grafts. It was seen that a close contact with the granules of the biomaterial resulted in thinning of the mucosa (<40 µm of width) and of the pseudostratified epithelium as well as perforations of the sinus mucosa. Both thinning and perforations of the mucosa increased between 2 and 10 weeks of healing.

The results are in agreement with other previous experimental studies in rabbits that showed similar damages to the sinus mucosa produced by biomaterials (13–15) or implants [13,16]. Similarly, in the present study, the thinned mucosa sites and the perforations were mostly located against sharpened edges of the biomaterial that were protruding beyond the dome shape of the elevated space. The results after 2 weeks cannot exclude that the pressure applied to the biomaterial during grafting might have influenced both perforations and thinning of the sinus mucosa. Nevertheless, the increased number of thinning sites and perforations over time related to the tendency of the sinus to regain its dimensions [19,20,21]. This will result in a forced repositioning of the sinus mucosa onto the elevated space, thoroughly delimiting its outline. It is on the periphery of the elevated space that the sinus mucosa will result in contact with graft. Especially when the granules’ projections protrude beyond the outline of the elevated space, the mucosa might be involved in the thinning process. The tissues compressed against the edges of the granules will react over time, starting with a displacement of vessels and mucosa glands, followed by thinning of the pseudostratified epithelium with loss of globet cells and cilias. Eventually, the mucosa will be perforated. Interestingly, at the perforation sites, the sinus mucosa appears to surround the exposed surface with a tapered epithelium completely. This represents a physiological reaction of the mucosa aiming to maintain secured the internal environment, as already described in previously published articles describing the effect on sinus mucosa of biomaterials [13,14,15] and implants [13,16]. However, with the progression of the exposure, the sinus mucosa will grow around the graft to complete its expulsion and regain continuity behind the granule.

The results from the present study showed that, after 10 weeks, the number of thinning sites and perforations was higher for the Xenogeneic group compared to the Synthetic group. This might be related to the different conformation of the surface of the granules, but also the way that the granules are resorbed or integrated into the newly form tissues within the elevated space. The xenogeneic graft used in the present study remains almost not resorbed during the first period of healing so that new bone will grow onto its surface [22,23,24,25], resulting in an osseointegration of the graft. The synthetic material used is instead partly resorbed and partly penetrated by newly formed bone. This event will conduce to the formation of a composite mixture of graft residual and bone that has been called “interpenetrating bone network” [18]. Both factors described above might have offered a certain degree of protection to the synthetic material from thinning and perforation events. Another experiment showed the importance of the biomaterial characteristics on the sinus mucosa damaging. Either autogenous bone or a xenogeneic graft was used for sinus augmentation in rabbits [13]. After 40 days of healing, only one thinning mucosa site and no perforations were detected in the autogenous group. Conversely, in the xenogeneic group, 96 thinned sites and three perforations in two sinuses out of six were observed.

It has to be considered that the corticalization of the new sinus floor subjacent the elevated sinus mucosa was not observed yet after 10 weeks. The formation of this layer of corticalized bone might protect from further perforations. In humans, a total or partial corticalization have been verified in 30% to 75% of cases after 9 months of healing [26,27,28,29].

The limitations of the present study are related to the smaller dimensions and thinner mucosa in rabbits compared to humans. The grafts appear huge in relation to the dimensions and the mucosa presents a width of ~80 µm. A histological analysis in human reported widths ranging between 0.45 and 1 mm [30]. A CBCT analysis [31] on eighty-eight sinuses programmed for sinus floor elevation reported a mean width of the sinus mucosa of 2 mm. However, thirty sinuses presented a width < 1 mm, the thinnest being 0.4 mm. It cannot be excluded that, over time, the thinnest sinus mucosa in human might also be damaged by the contact to sharpen projections of not resorbed grafts, resulting eventually in perforations. The sinus mucosa showed repairing processes around the exposed graft that might result in the expulsion of the graft into the sinus cavity with complete restoration of the continuity of the sinus mucosa. The granule might be eliminated through the ostium/infundibulum preventing infective sinusal problems. The extrusion of biomaterial into the sinuses has been reported both during the surgical procedure [6,7] or at a later stage requiring the removal of the graft sometimes [11,32]. Complications might be associated with sinus floor elevation [33]. Short implants have been shown to represent a valid alternative, exhibiting lower marginal bone loss compared to standard-length implants and similar survival rate [34].

## 5. Conclusions

In conclusion, the contact with xenogeneic or synthetic grafts will induce thinning and possible perforation of the sinus mucosa. This effect will increase over time, and it is stronger at the xenogeneic than the synthetic graft.

## Data Availability

The data are available on reasonable request.
