# Correction: Omori et al. Sinus Mucosal Damage Triggered by Synthetic or Xenogeneic Bone Substitutes: A Histological Analysis in Rabbits. *J. Funct. Biomater.* 2022, *13*, 257

**DOI:** 10.3390/jfb15050121

**Published:** 2024-05-06

**Authors:** Yuki Omori, Daniele Botticelli, Stefano Migani, Vitor Ferreira Balan, Eduardo Pires Godoy, Samuel Porfirio Xavier

**Affiliations:** 1 Department of Oral Implantology, Osaka Dental University, 8-1 Kuzuhahanazonocho, Osaka 573-1121, Japan; info@omori-dent.com; 2 ARDEC Academy, Viale Giovanni Pascoli 67, 47923 Rimini, Italy; dott.miganistefano@gmail.com; 3 Department of Oral and Maxillofacial Surgery and Periodontology, Faculty of Dentistry of Ribeirão Preto, University of São Paulo, Av. do Café-Subsetor Oeste-11 (N-11), Ribeirão Preto 14040-904, Brazil; vitor.balan@usp.br (V.F.B.); spx@forp.usp.br (S.P.X.); 4 Department of Oral Biology, Faculty of Dentistry of Ribeirão Preto, University of São Paulo, Ribeirão Preto 14040-904, Brazil; eduardo.godoy@usp.br

## Error in Figure

In the original publication [1], there was a mistake in Figure 1 with regards to what was published. Figure 1a is wrong, as it is just a repetition of Figure 2a. The correct version of Figure 1a appears below. 


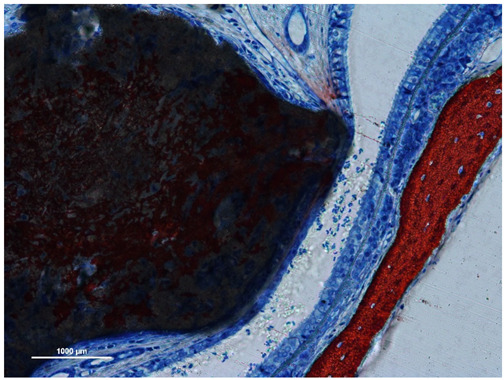


## Figure Legend

In the original publication [1], there was a mistake in the legend for Figure 1. The legend of Figure 1a was wrong because it did not describe the correct figure that should have been used. The correct legend appears below.

**Figure 1**. (**a**) Synthetic site: Stevenel’s blue and alizarin red stain. (**b**) Xenogeneic site: toluidine blue stain. Note the progressive decrease in width of both sinus mucosae and pseudostratified epithelia. A loss of cilia is evident in the thinnest sites on both biomaterials. While the process of resorption has a minimal impact on the xenogeneic graft, the synthetic graft has undergone a process already described as an interpenetrating bone network [18] characterized by concurrent bone formation within the biomaterial structure during its resorption.

The authors state that the scientific conclusions drawn in the paper are unaffected. This correction was approved by the Academic Editor. The original publication has also been updated.
